# Xanthine oxidase inhibition by medicinal plants mitigates tenofovir-induced nephrotoxicity: a comprehensive computational and experimental study

**DOI:** 10.3389/fphar.2026.1775162

**Published:** 2026-04-21

**Authors:** Chaitanya Sankannavar, Harish R. Darasaguppe, Vishal S. Patil, Faizan A. Beerwala, Swarup S. Gujarathi, Rajitha Charla, Priyanka P. Patil, Vishwambhar V. Bhandare, Nayeem A. Khatib, Subarna Roy

**Affiliations:** 1 Department of Microbiology and Molecular Biology, Indian Council of Medical Research-National Institute of Traditional Medicine, Belagavi, Karnataka, India; 2 Department of Pharmacology, KLE College of Pharmacy, Belagavi, KLE Academy of Higher Education and Research (KAHER), Belagavi, Karnataka, India

**Keywords:** *Allium cepa*, nephrotoxicity, reverse transcriptase inhibitor, tenofovir, *Vitex negundo*, xanthine oxidase

## Abstract

**Background:**

Tenofovir, a nucleotide reverse transcriptase inhibitor (NRTI), is a first-line therapy for HBV and HIV and is administered as tenofovir disoproxil fumarate (TDF) or tenofovir alafenamide (TAF). However, long-term use of TDF is linked to renal and other organ toxicities, which is largely attributed to oxidative stress mediated by xanthine oxidase (XO), a key enzyme in purine metabolism. To address this, plants with documented nephroprotective and XO-inhibitory properties were investigated through computational, *in vitro*, and *in vivo* approaches.

**Methods:**

Bioactive compounds from nine medicinal plants were subjected to computational screening for XO inhibition, leading to the selection of *Allium cepa* and *Vitex negundo* as promising candidates. Fifteen extracts from different plant parts were prepared and assessed *in vitro* for XO inhibitory activity. The methanolic extract of *A. cepa* bulb (AC) and the hydroalcoholic extract of *V. negundo* root (VN) demonstrated the highest potency. These extracts were further evaluated for their antioxidant potential and tested against TDF-induced renal and hepatic toxicities in a 35-day rat model.

**Results:**

Both AC and VN significantly mitigated TDF-induced elevations in serum biomarker levels, including creatinine, BUN, AST, ALT, GGT, bilirubin, and alkaline phosphatase, while enhancing albumin levels. They also restored the levels of oxidative stress markers such as MDA, GSH, catalase, and SOD. Histopathological examination confirmed substantial structural recovery of liver and kidney tissues.

**Conclusion:**

Overall, this preclinical study demonstrates that *A. cepa* and *V. negundo* extracts effectively alleviate TDF-induced organ toxicities and oxidative damage, supporting their potential as adjunct therapies to enhance the safety of TDF treatment in HBV and HIV management.

## Highlights

• Xanthine oxidase activity–inhibiting medicinal plants were evaluated for their protective effects against tenofovir-induced nephrotoxicity.
• The extracts of *Allium cepa* and *Vitex negundo* showed significant XO inhibitory and antioxidant activity.
• The plant extracts ameliorated organ toxicities and demonstrated nephroprotective effects *in vivo*.
• Computational studies predicted the compounds’ stability with XO for a 100-ns run.

## 1 Introduction

Chronic hepatitis B (CHB) viral infection is a global public health problem. The World Health Organization (WHO) estimates that 254 million people live with chronic hepatitis B (Easterbrook et al., 2024), leading to approximately 1.1 million deaths annually due to cirrhosis and liver cancer and 42.3 million people living with HIV, with 630,000 deaths and 1.3 million new infections annually (Tang et al., 2014). For the treatment of these infectious diseases, the NRTI oral prodrug of tenofovir, namely, tenofovir disoproxil fumarate (TDF), is the most widely prescribed for CHB and HIV infections due to its advantages over the other approved drugs (Nelson et al., 2007; de Clercq et al., 2010). However, it is costly and requires a high dose (300 mg/day) (Wassner et al., 2020).

Long-term use of TDF has been associated with various renal toxicities, including hypophosphatemia, proximal tubular injury, acute tubular necrosis, Fanconi syndrome, renal tubular dysfunction, proteinuria, aminoaciduria, glycosuria, enhanced neutrophil infiltration, apoptosis, mitochondrial damage, inducible nitric oxide synthase (iNOS) activation, increased oxidative stress, reduced antioxidant enzymatic activity, and activation of NF-κB-mediated inflammatory pathways (Nelson et al., 2007; Ramamoorthy et al., 2021). Furthermore, it induces hepatic injury through mitochondrial DNA (mtDNA) depletion, mitochondrial dysfunction, and elevated mitochondrial reactive oxygen species (mtROS), ultimately contributing to hepatotoxicity (Vazi et al., 2023). In addition, it has been linked to bone demineralization and osteomalacia, primarily due to phosphate wasting and mitochondrial dysfunction in the osteoblasts (Barbieri et al., 2018). In addition, metabolic disturbances, including dyslipidemia and lactic acidosis, have been reported in some patients receiving prolonged therapy (Jung et al., 2017).

TDF first undergoes metabolization to produce tenofovir after being taken up by cells. Tenofovir is phosphorylated from its drug form to produce its active metabolites, which are mono, diphosphate (DP), or triphosphate (TP). Xanthine oxidase (XO) is the key enzyme involved in the metabolism of nucleoside(tide) analogs (Cicero et al., 2021). Its catalyzes the aerobic dehydrogenation of purine hypoxanthine to uric acid and produces ROS as by-products under normal physiological conditions (Nishino and Okamoto, 2015; Hsieh et al., 2007). It is expressed in various body cells such as the liver, muscle, abdomen, lungs, kidneys, cardiac, and brain (Borges et al., 2012). The generation of ROS by XO has been linked to lipid peroxidation, membrane disruption, and tissue injury. Oxidation of proteins and nucleic acids leads to organ damage and cardiovascular disorders. The XO inhibitor allopurinol is the drug of choice for gout and various kidney diseases (Pacher et al., 2006). However, it is also associated with maculopapular pruritic rash, gastrointestinal adverse effects, nephrolithiasis, allopurinol hypersensitivity syndrome, Stevens–Johnson syndrome, renal toxicity, allergic reactions, and fatal liver necrosis (Taejarernwiriyakull et al., 2011).

Several plants have been reported to possess nephroprotective and XO-inhibitory activities, such as *Allium cepa* (Nile and Park, 2013; Ouyang et al., 2017), *Asparagus racemosus* (Nile and Park, 2014), *Coccinia grandis* (Umamaheswari and Chatterjee, 2008; Satrasala et al., 2021), *Moringa oleifera* (Edeogu et al., 2020; Tian et al., 2021), *Tinospora cordifolia* (Uppuluri et al., 2013), *Trachyspermum ammi* (Bilal et al., 2019; Ishaq et al., 2015), *Vitex negundo* (Janakiraman et al., 2015; Umamaheswari et al., 2007), *Gloriosa superba* (Rahaman et al., 2022), and *Piper betel* (Murata et al., 2009). Therefore, we hypothesized that concomitant administration of selected medicinal plant extracts with TDF may reduce drug excretion, enhance bioavailability, and mitigate TDF-induced nephrotoxicity and other organ toxicities through XO inhibition. Accordingly, the present study was designed to identify potential XO inhibitors from these medicinal plants and evaluate their protective effects against TDF-induced renal and hepatic toxicities in laboratory animal models.

## 2 Materials and methods

### 2.1 *In silico* pharmacology

#### 2.1.1 Selection of traditional medicinal plants

Nine traditional medicinal plants, namely, *A. cepa, A. racemosus, C. grandis, M. oleifera, T. cordifolia, T. Ammi, V. negundo*, *G. superba*, and *P. betel*, were selected for their XO inhibition and nephroprotective activity based upon findings from previous studies.

#### 2.1.2 Mining of phytocompounds

Phytocompoundsfrom the selected plants were listed using published literature and retrieved using publicly available small-molecule databases such as Dr. Dukes Database (https://phytochem.nal.usda.gov/) and IMMPAT database (https://cb.imsc.res.in/imppat/). The compiled list was further curated to include only pharmacologically active compounds. The PubChem chemical database (https://pubchem.ncbi.nlm.nih.gov/) was used to obtain chemical information such as molecular formula, molecular weight, and simplified molecular-input line-entry system (SMILES).

#### 2.1.3 Molecular docking studies

The 3D structures of the selected phytocompounds and the standard XO inhibitor allopurinol were retrieved from the PubChem database (https://pubchem.ncbi.nlm.nih.gov) in “.sdf” format and converted to “.pdb” format using Discovery Studio Visualizer 2019 (https://discover.3ds.com/discoverstudio-visualizer-download). The XO protein structure (PDB ID: 3AM9) was obtained from the Protein Data Bank (RCSB, www.rcsb.org). Prior to docking, all heteroatoms and pre-bound ligands were removed from the protein, and the structure was refined and saved in “.pdb” format. All the ligands were subjected to energy minimization using the MMFF94 force field in Open Babel, and the lowest-energy conformation was selected for docking. Molecular docking was performed using AutoDock 4.2, where Kollman charges and hydrogen atoms were added to the protein. The Lamarckian genetic algorithm was employed for docking after defining the grid box. Following docking, the optimal ligand pose was selected based on the lowest binding energy from the .dlg output file. Protein–ligand interactions were visualized using Discovery Studio Visualizer. Additionally, the PrankWeb server (https://prankweb.cz/), was used to predict the ligand-binding sites and their active residues, as previously reported (Beerwala et al., 2024).

#### 2.1.4 Stability of the docked complex

GROMACS 2021.3 (http://www.gromacs.org/) was used to perform molecular dynamics (MD) simulations to evaluate the structural stability of the XO protein in the complex with the selected docked compounds and the standard drug for a period of 100 ns. The Amber ff99SB-ildn force field was applied to the protein, and the ligand partial charges were generated using Antechamber with the “bcc” charge model. The XLeap module from AmberTool was used to generate ligand complexes enclosed inside a solvated rectangular box with dimensions of 10.0 Å from the protein border in all directions (Patil et al., 2025). An adequate number of NA^+^/CL^–^ counter-ions balanced the simulated system. The steepest descent and conjugate gradient approaches produced conformations with the lowest energy levels close to the global minimum state (DasNandy et al., 2022; Beerwala et al., 2025). The system was equilibrated using the NVT and NPT ensembles for 1 nanosecond to ensure constant atom count, volume, temperature, and pressure. The examination was conducted at a temperature of 300 K and a pressure of 1 bar. PME managed Coulomb electrostatic interactions, whereas LINCS constrained H-bonds. The system conducted a 100-nanosecond molecular dynamics test. The trajectories were assessed using GROMACS’ internal tools “gmx rms,” “rmsf,” “hbond,” “gyrate,” and “sasa.”

### 2.2 *In vitro* pharmacology

#### 2.2.1 Plant material collection, authentication, and extraction

Based upon *in silico* findings, two medicinal plants, *A*. *cepa* (bulb and outer skin) and *V*. *negundo* (roots, seeds, and leaves), were prioritized. The plant material of *V. negundo* was collected from the botanical garden of ICMR–National Institute of Traditional Medicine (NITM), Belagavi, whereas *A. cepa* bulb was procured from the local market of Belagavi, Karnataka, India. These plant parts were authenticated by the plant taxonomist at ICMR–NITM, Belagavi, and voucher specimens were deposited at the institute herbarium with accession numbers RMRC-1679 (*A. cepa*) and RMRC-1680 (*V. negundo*) for future reference.

The collected plant materials were washed under running water to remove impurities, chopped, shade-dried, and pulverized into coarse powder. Ethanolic and methanolic extractions were performed by maceration at ambient temperature for 7 days, while hydroalcoholic extractions were carried out using a Soxhlet apparatus (León-Roque et al., 2023; Rajesh et al., 2023) (as detailed in Supplementary Material File S1), yielding a total of 15 extracts (five plant parts in three different solvents). The filtrates obtained were concentrated using a rotary evaporator at 40 °C, followed by lyophilization to obtain dry extracts, which were stored at −20 °C in an amber-colored bottle until further use. For experimental studies, stock solutions of the dried extracts were prepared at a concentration of 10 mg/mL in 5% (v/v) DMSO diluted with sterile water and sterilized using a 0.22 µm syringe filter. 

#### 2.2.2 XO-inhibitory activity assay

The XO-inhibitory activities of 15 prepared extracts and the standard drug allopurinol were evaluated using a commercial XO assay kit [Abcam (ab102522), Cambridge, MA, United States] as per the manufacturer’s instructions, with minor modifications. Briefly, 50 μL each of different concentrations of the test samples (500, 250, and 125 μg/mL) were added to 50 μL of the reaction mixture containing the rat liver homogenate supernatant as a source of XO enzyme. Immediately after adding the mixture (A1) and after 20 min of incubation, the absorbance at 570 nm was measured spectrophotometrically (A2). The A2 reading was subtracted from the A1 reading to remove the background (ΔA = A2 – A1).

$$\%\text{ Inhibition}=\left(\left(\Delta A-\Delta B\right)\right)/\left(\Delta A\right)\;X\;100,$$

where A represents the enzymatic activity in the liver homogenate in the absence of the test extract and B represents the test extract with the enzyme and reaction mixture, respectively. The results were expressed in % of inhibition by the test samples in comparison with the enzymatic activity in the liver homogenate alone.

#### 2.2.3 *In vitro* antioxidant activity assays

##### 2.2.3.1 DPPH radical scavenging assay

The free radical scavenging activity of the plant extracts was determined using 2,2-diphenyl-1-picrylhydrazyl (DPPH), following the method described by Hussen and Endalew (2023) with slight modifications. This assay is based on the ability of antioxidants to scavenge DPPH radicals by donating hydrogen atoms, resulting in a color change from purple to yellow and a corresponding decrease in the absorbance. The DPPH reagent was prepared by dissolving 0.004 g of DPPH in 100 mL of methanol to obtain a 0.1 mM solution. A stock solution of each plant extract (10 mg/mL) was prepared and further diluted in 70% ethanol to achieve concentrations ranging from 7.81 to 250 μg/mL. For the assay, 100 µL of each diluted extract and ascorbic acid was mixed with 1 mL of the DPPH solution in microcentrifuge tubes and incubated in the dark at room temperature for 30 min. The absorbance was measured at 517 nm using a microplate UV–Vis spectrophotometer. Methanol served as the blank, and ascorbic acid in methanol was used as the standard, with the concentration ranging from 1 to 60 μg/mL. The percentage of DPPH radical scavenging was calculated, and the IC_50_ value, representing the concentration at which 50% of DPPH radicals were scavenged, was determined for each extract.

$$\text{Scavenging }\left(\%\right)=\frac{\left(\text{Absorbance}\;\text{of}\;\text{blank}-\text{Absorbance}\;\text{of}\;\text{sample}\right)}{\text{Absorbance}\;\text{of}\;\text{blan}k}\times 100.$$

##### 2.2.3.2 Nitric oxide (NO) assay

Nitric oxide (NO) plays a key role in oxidative stress and inflammation, and its scavenging by plant extracts is an important indicator of antioxidant potential. The NO produced from sodium nitroprusside (SNP) was measured using the method of Marcocci et al. (1994) with slight modifications. Briefly, the Griess reagent was prepared by mixing 0.1% N-(1-naphthyl)ethylenediamine dihydrochloride and 1% sulfanilamide in 2.5% phosphoric acid. For the assay, 1 mL of varying concentrations of the plant extract (31.25 μg/mL–1,000 μg/mL) was mixed with 0.5 mL of 10 mM SNP in phosphate-buffered saline (PBS) and incubated at 25 °C for 180 min. An equal volume of freshly prepared Griess reagent was added after incubation. The reaction mixture was then transferred to a 96-well microplate, and the absorbance was measured at 546 nm using a microplate UV–Vis spectrophotometer. Ascorbic acid served as the positive control. The percentage of NO scavenging was calculated relative to the control.

## 3 *In vivo* pharmacology

Adult male Sprague–Dawley rats (180 g–200 g) were procured from *In Vivo* Biosciences, and the standard pellet feed was obtained from Champaka Feeds and Foods. The animals were housed in standard polypropylene cages under controlled laboratory conditions (23 °C ± 2 °C, 12-h light/12-h dark cycle, and 50%–60% relative humidity) with *ad libitum* access to food and water. The animals were acclimatized for 7 days before experimentation. The animal study was approved by the Institutional Animal Ethics Committee (IAEC) of KLE College of Pharmacy, Belagavi (Reg. No. 221/Po/Re/S/2000/CPCSEA) in its meeting dated 21/12/2021 (Resolution No. 31), following the CCSEA guidelines.

Animal grouping and experimentation: Forty-five male Sprague–Dawley rats weighing 180 g–200 g were randomly assigned into nine groups (n = 5). All animals, except those in the normal group, were orally administered TDF (600 mg/kg) p.o. for 35 days to induce nephrotoxicity (Ramamoorthy et al., 2019; Ramamoorthy et al., 2021). The grouping and treatment details were as follows: normal group: received drinking water orally (p.o.); TDF group: received TDF (600 mg/kg) in sterile water, p.o.; AP10 + TDF: received allopurinol (10 mg/kg) + TDF (600 mg/kg), p.o; AC 100 + TDF: received AC (100 mg/kg), p.o. + TDF (600 mg/kg), p.o; AC 200 + TDF: received AC (200; mg/kg), p.o. + TDF (600 mg/kg), p.o; AC 400 + TDF: received AC (400 mg/kg), p.o. + TDF (600 mg/kg), p.o; VN 100 + TDF: received VN (100 mg/kg), p.o. + TDF (600 mg/kg), p.o; VN 200 + TDF: received VN (200 mg/kg), p.o. + TDF (600 mg/kg), p.o; VN 400 + TDF: received VN (400 mg/kg), p.o. + TDF (600 mg/kg), p.o. All treatments were co-administered once daily for 35 days along with TDF. Throughout the study period, the body weight, food intake, and water intake of each animal were recorded daily. At the end of the experiment, urine and blood samples were collected for the estimation of biochemical parameters. Subsequently, the animals were euthanized using an overdose of ketamine. The liver and kidney were harvested and weighed to determine organ weight variations. Portions of liver and kidney tissues were homogenized for antioxidant assays, while the remaining tissues were fixed in 10% neutral buffered formalin solution for histopathological analysis.

### 3.1 Serum biochemical parameters

At the end of the experiment, blood samples were collected from each rat via the retro-orbital plexus and cardiac puncture. Serum levels of liver function markers, including aspartate aminotransferase (AST; BioSystems #11830), alanine aminotransferase (ALT; BioSystems #11830), alkaline phosphatase (ALP; BioSystems #11592), and albumin (ALB; BioSystems #11547), along with renal function markers such as serum creatinine (BioSystems # 12502) and blood urea nitrogen (BUN; BioSystems #11516), were analyzed using a semi-automated analyzer (A15, BioSystems S.A., Barcelona, Spain).

### 3.2 Quantification of enzymatic and non-enzymatic antioxidant biomarkers in the liver and kidney

The liver and kidney were dissected and weighed, and 200 mg of each organ was homogenized in 10 mL of cold phosphate buffer (pH 7.4). The homogenates were centrifuged at 12,000 x g for 10 min. The supernatant was then subjected to a second centrifugation at 20,000 x g for 1 h at 4 °C. The supernatant was used for the quantification of antioxidant biomarkers, including superoxide dismutase (SOD), catalase (CAT), glutathione (GSH), and lipid peroxidase (LPO). Briefly, GSH levels were measured by incubating 250 µL of the liver homogenate with 50 µL of DTNB and 2.5 mL of sodium phosphate buffer for 30 min, followed by measuring the absorbance at 412 nm. SOD activity was determined by adding 25 µL of the homogenate to 50 µL of epinephrine (0.1 mM in carbonate buffer) and recording the change in absorbance at 295 nm between 60 and 120 s. Lipid peroxidation was assessed by incubating 200 µL of the liver homogenate at 95 °C for 1 h with a mixture containing 1.5 mL of thiobarbituric acid (0.8% w/v), 20% acetic acid, and 200 µL of sodium lauryl sulfate (8.1% w/v). The samples were cooled and centrifuged at 3,000 x g for 10 min, and the absorbance of the organic layer was recorded at 532 nm (Patil et al., 2023).

### 3.3 Histopathological evaluation of liver and kidney tissues

Two tissue samples of each organ (i.e., the liver and kidney) of each rat were fixed in 10% neutral buffer formalin for histopathological analysis. The tissues were processed, sectioned, and stained with eosin–hematoxylin (H&E) dye. The stained sections were examined under a light microscope at ×40 magnification to evaluate the histopathological changes (Patil et al., 2023; Taiwo Oluwaloye et al., 2023).

## 4 Statistical analysis

The results are presented as mean ± SEM. Differences between group means were evaluated using one-way ANOVA followed by Tukey’s *post hoc* test with GraphPad Prism version 10.6.1. Statistical significance was defined as p < 0.05. The network was assessed using “Edge count,” and the docking score of binding energies (BE) is represented in kcal/mol.

## 5 Results

### 5.1 *In silico* pharmacology

#### 5.1.1 Identification of phytocompounds

A total of 145 pharmacologically active phytocompounds were shortlisted from nine selected medicinal plants through the screening of published literature and publicly available databases (Supplementary Material File S2).

#### 5.1.2 Molecular docking study

**FIGURE 1:**
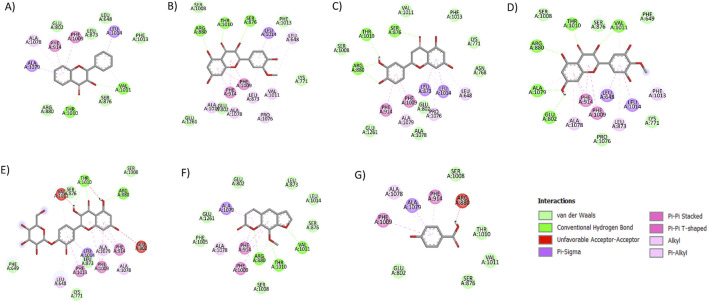
2D interactions of **(A)** dihydroflavanol, **(B)** isorhamnetin, **(C)** spiraeoside, **(D)** luteolin, **(E)** marnsetin, **(F)** xanthotoxin, and **(G)** allopurinol with the XO protein. All the H-bond interactions are presented in green, whereas the hydrophobic interactions are presented in other colors other than green.

Molecular docking of 145 phytocompounds from nine medicinal plants with XO revealed that compounds from *A. cepa* and *V. negundo* showed the lowest BEs (Supplementary Material File S3). Among the top-10 hits, three compounds from *A. cepa* and three from *V. negundo* showed the highest affinity for the XO active site (Supplementary Material File S4). From *A. cepa*, dihydroflavonol showed the lowest BE of −9.9 kcal/mol, forming two hydrogen bonds, i.e., Arg880 … O and Arg880 … OH, and five nonhydrogen bonds via Arg880, Thr1010, Ala1079, and Phe914 (2). Isorhamnetin exhibited a BE of −9.5 kcal/mol, with three hydrogen bonds and seven nonhydrogen bonds, while spiraeoside had a BE of −8.9 kcal/mol, forming two hydrogen bonds and 14 nonhydrogen bonds. Among these, two interactions spanned the active site residues. From *V. negundo*, the top-three compounds were predicted to form stable interactions with active site residues. Luteolin exhibited the lowest BE of −10.1 kcal/mol, forming three hydrogen bonds, i.e., Arg880 … O, Thr1010 … = O, and Ser876 … O, and 13 nonhydrogen bonds via Ala1079, Ala1078, Phe914 (2), Phe1009 (2), Leu873, Pro1076, Val1011, Leu648 (2), and Leu1014 (2). Mearnsetin showed a BE of −9.0 kcal/mol, forming four hydrogen bonds, i.e., Arg880 … O (2), Thr1010 … O, and Val1011 … O, and eight nonhydrogen interactions via Val1011, Arg880, Phe914 (2), Phe1009, Ala1078, and Ala1079 (2). Similarly, xanthotoxin had a BE of −8.9 kcal/mol, forming four hydrogen bonds, i.e., Thr1010 … OH, Ser876 … O, Arg880 … OH, and Arg880 … O, and eight nonhydrogen bonds via Phe914, Phe1009, Ala1079, Pro1076, Leu873 (2), Leu1014, and Leu648. Three of these interactions spanned the active site residues (Figure 1). The standard XO inhibitor allopurinol had a BE of −6.3 kcal/mol, forming four hydrogen bonds, i.e., Ala1079 … = O, Glu802 … H, Glu802 … N, and Thr1077 … NH, and nine nonhydrogen bonds via Phe914 (2), Phe1009 (2), Ala1078, Arg880, and Ala1079 (3).

#### 5.1.3 Stability of the protein–ligand complexes

Based on the lowest BE and maximum intermolecular interactions with active site residues, three compounds were prioritized for MD simulation, i.e., mearnsetin and luteolin form *V. negundo*, isorhamnetin from *A. cepa*, and allopurinol as a standard comparator molecule. The stability of complex formation of the XO with the compounds was validated by 100-ns MD simulations. Furthermore, to evaluate the structural stability of XO in conjunction with the ligands, we analyzed key parameters, including the root mean square deviation (RMSD), root mean square fluctuation (RMSF), radius of gyration (Rg), solvent-accessible surface area (SASA), and hydrogen-bond interactions.

##### 5.1.3.1 RMSD analysis of the complexes

**FIGURE 2:**
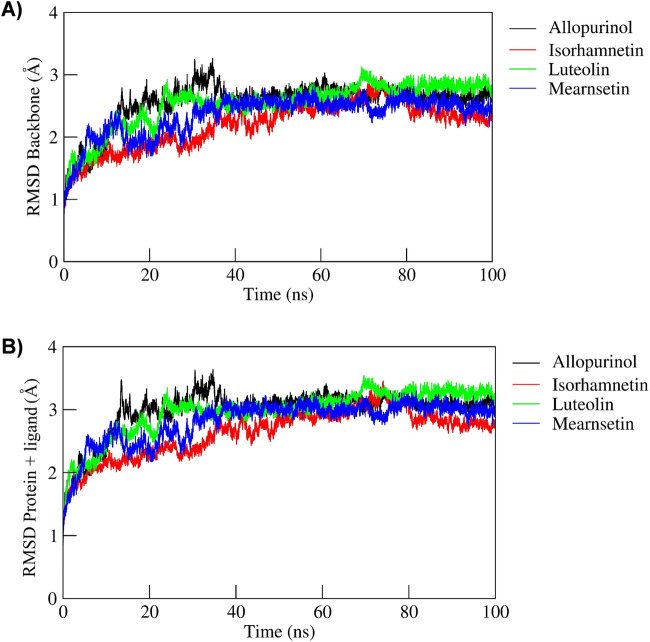
Root mean square deviation plot for **(A)** backbone and **(B)** Protein + Ligand atoms of complexes over the 100-ns MD simulation run.

**FIGURE 3:**
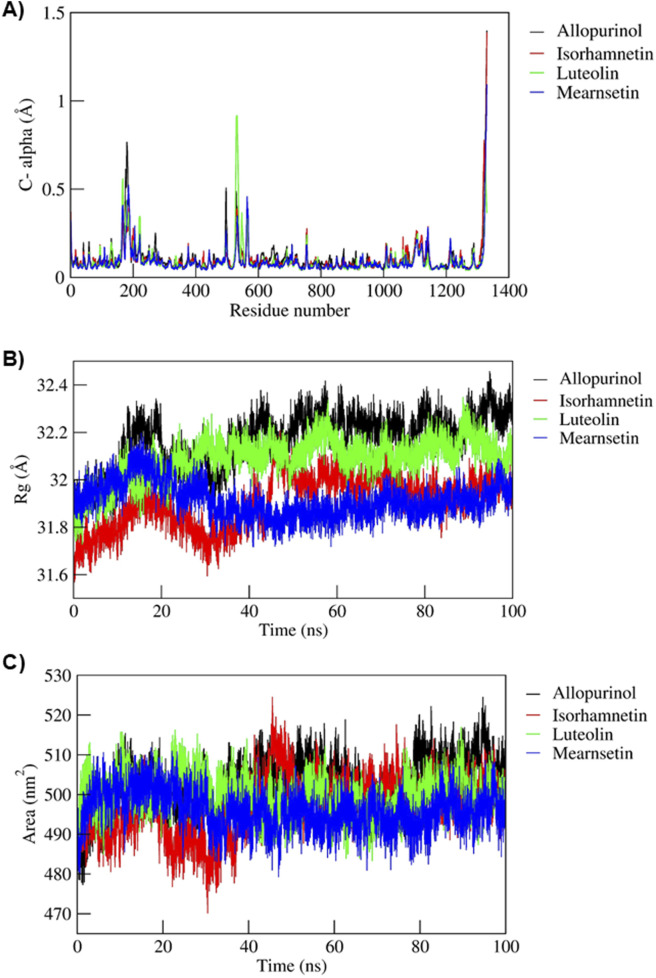
**(A)** Root mean square fluctuations plot for C-alpha atoms, **(B)** radius of gyration, and **(C)** solvent-accessible surface area of protein-–ligand complexes over the 100-ns MD simulation run.

RMSD is used to measure the differences in the backbone atoms of a protein as it transitions from its initial conformation to its final position during simulations. The stability of the protein complex can be assessed based on the magnitude of these deviations; smaller deviations indicate greater conformational stability of the complex throughout the simulation. Figure 2 shows the RMSD values of the backbone atoms across the trajectories, indicating the steady dynamics of all the simulated ligand complexes. As shown, the backbone atoms’ RMSD values of XO with allopurinol (black), mearnsetin (blue), and luteolin (green) show moderate fluctuations up to 35 ns. Similarly, the RMSD values of XO complexed with isorhamnetin (red) showed a minor fluctuation at 40 ns. The average backbone RMSD values of allopurinol, mearnsetin, and luteolin were observed as ∼1.26 Å, ∼1.23 Å, and ∼1.25 Å, respectively. On the other hand, the average RMSD of isorhamnetin was observed to be ∼1.13 Å. Furthermore, the comparison between the initial and final structures from the MD simulation revealed minimal fluctuations in the RMSD of the structure throughout the simulation, i.e., allopurinol (∼0.75 and ∼1.26), isorhamnetin (∼0.63 Å and ∼1.22 Å), luteolin (∼0.63 Å and ∼1.27 Å), and mearnsetin (∼0.72 Å and ∼1.24 Å). Furthermore, we analyzed the RMSD fluctuations for the complexes (ligand-bound), and a similar trend was seen as that of the RMSD backbone. However, the significant change in allopurinol was seen due to the structural alteration in the complex at 15 ns–20 ns and later at 25 ns–40 ns, after which the complex attains steady RMSD values. Thus, in general, all the five complexes express stable dynamics during the 100-ns MD simulation with the RMSD fluctuations of <4 Å, respectively, as shown in Figure 3.

##### 5.1.3.2 RMSF analysis of the complexes

The RMSF values of all the simulated complexes are shown in Figure 3A. Notably, the N-terminal region consistently exhibited a higher degree of fluctuation than other regions of the protein. A closer examination of the 3D structure revealed increased conformational flexibility with stable interactions. The residues contributing to binding within the pocket cavity maintained interactions within a distance cutoff of <2 Å. Moreover, other flexible loops of RMSD values were observed at the N-terminal. Due to the maximal residual fluctuations at ∼1.8 Å, ∼1.1 Å, and ∼0.97 Å, a greater peak was observed between aa190 and aa205, aa410 and aa420, and aa690 and aa695 in the allopurinol complex, as shown in. Similarly, multiple peaks of moderate-to-high fluctuations were observed in the mearnsetin complex with the maximum of ∼1.32 Å and ∼0.47 Å respectively. Additionally, seven moderate fluctuations were observed between aa185 and aa210, aa425 and aa430, aa470 and aa510, and aa1140 and aa1170. Four peaks were observed in the luteolin complex: one between aa175 and aa190 at ∼0.61 Å, the second between aa210 and aa220 at ∼0.37 Å, the third between aa570 and aa585 at ∼0.94 Å, and the last between aa760 and aa785 at ∼0.28 Å. Three moderate peaks at ∼0.58 Å, ∼0.47 Å, and ∼0.31 Å were observed in the isorhamnetin complex, one of which was aa190 to aa200, followed by aa470 to aa490, and, finally, aa760 to aa775, respectively.

##### 5.1.3.3 Rg and SASA analysis

The Rg serves as a key indicator of protein folding and structural compactness, primarily reflecting the conformation of the protein backbone. To assess the compactness of the complexes, Rg and SASA analysis was conducted, with the results presented in Figure 3B. Overall, the Rg values of the simulated complexes exhibited stable behavior throughout the MD simulations, except for the allopurinol complex. Specifically, the Rg values of the allopurinol complex showed a notable and consistent increase at approximately ∼20 ns, followed by stabilization from ∼40 ns until the end of the simulation at 100 ns. In contrast, the other complexes—including isorhamnetin, luteolin, and mearnsetin—maintained relatively stable Rg values throughout the simulation, ranging between approximately ∼31.4 Å and ∼32.31 Å.

Furthermore, as shown in Figure 3C, the SASA values for all the analyzed complexes indicate significant structural stability, supporting the formation of compact globular shapes throughout the 100-ns simulation period. On average, the SASA values ranged from 527.36 nm^2^ to 468.23 nm^2^. Specifically, the initial and final SASA values were recorded as follows: allopurinol (484.07 nm^2^ and 509.45 nm^2^), isorhamnetin (481.23 nm^2^ and 497.00 nm^2^), luteolin (488.65 nm^2^ and 498.31 nm^2^), and mearnsetin (484.04 nm^2^ and 494.70 nm^2^). These findings emphasize the stable formation and maintenance of the complexes over the course of the simulation.

##### 5.1.3.4 Hydrogen bond interactions

**FIGURE 4:**
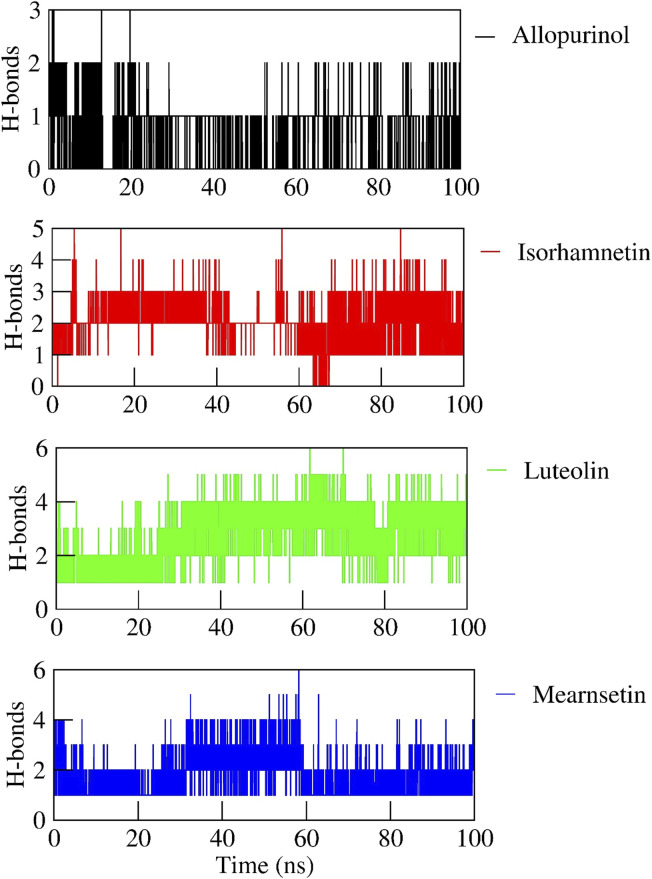
Hydrogen-bond interaction formed between protein and ligand complexes in the 100-ns MD simulation run.

The hydrogen bond interactions between the protein and ligand complexes were monitored throughout the 100-ns simulation (Figure 4). Overall, the native nonbound contacts remained consistently stable during the simulation, indicating robust complex formation across all the simulated systems. The allopurinol complex formed a total of three hydrogen bonds, with two remaining consistent throughout the entire simulation run. Likewise, the isorhamnetin complex formed five hydrogen bonds, of which, four were consistent. Meanwhile, six hydrogen bonds each were formed in the luteolin and mearnsetin complexes, and five hydrogen bonds were seen to be consistent throughout the simulation.

### 5.2 *In vitro* pharmacology

#### 5.2.1 Xanthine oxidase inhibition assay

**FIGURE 5:**
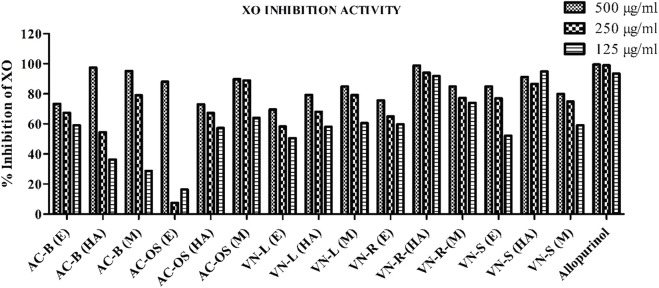
Inhibition of nitric oxide radicals by *A. cepa* and *V. negundo* extracts evaluated by the DPPH assay. Data are presented as mean ± standard deviation in triplicates.

Based upon the *in silico* studies, the *A. cepa* and *V. negundo* phytoconstituents showed the most favorable binding with the active site of XO. Fifteen different extracts from different parts, i.e., *A. cepa* bulb and outer skin and *V. negundo* root, seed, and leaves, were prepared using three different solvents (methanol, ethanol, and 70% ethanol in water (v/v)). Different levels of XO inhibition by three different concentrations (500 μg/mL, 250 μg/mL, and 125 μg/mL) of these extracts were observed, and allopurinol, a standard XO inhibitor, was used as the positive control. As shown in Figure 5, among the 15 extracts, the hydroalcoholic root extract of *V. negundo* (VN) showed the highest XO inhibitory activity at 98.88%, followed by the methanolic bulb extract of *A. cepa* (AC), with 97.45% inhibitory activity at 500 μg/mL. Detailed inhibition data for all the extracts are provided in Supplementary Material File S5.

#### 5.2.2 DPPH and NO free radical scavenging activity

**FIGURE 6:**
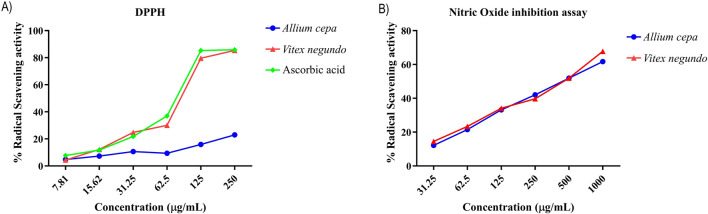
**(A)** Antioxidant and **(B)** inhibitory activities of nitric oxide radicals by the *A. cepa* and *V. negundo* extracts evaluated by the DPPH assay. Data are presented as mean ± standard deviation in triplicates.

For the DPPH assay, the IC_50_ values of AC and VN were 517.79 μg/mL and 119.86 μg/mL, respectively, whereas the IC_50_ of the standard comparator, ascorbic acid, was 81.74 μg/mL (Supplementary Material File S6). For the NO assay, the IC_50_ values of AC and VN were 625.40 μg/mL and 577.05 μg/mL, respectively, while the standard ascorbic acid had an IC_50_ value of 31.92 μg/mL (Figures 6A,B).

### 5.3 *In vivo* pharmacology

#### 5.3.1 Physical parameters

##### 5.3.1.1 Percentage change in the body weight, food intake, and water intake

**FIGURE 7:**
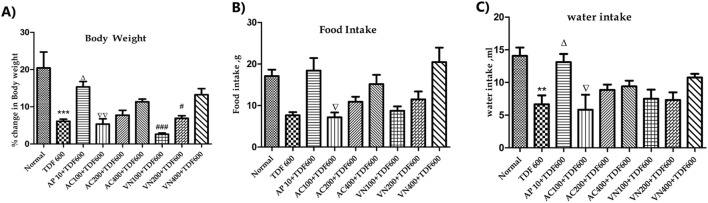
Effects of AP, AC, and VN on % change in the **(A)** body weight, **(B)** food intake, and **(C)** water intake. All the values are expressed as mean ± SEM (n = 5). One-way analysis of variance (ANOVA) followed by Tukey’s test. Statistical significance is indicated as *p < 0.05, **p < 0.01, and ***p < 0.001 compared to normal; Δp < 0.05, ΔΔp < 0.01, and ΔΔΔp < 0.001 compared to TDF.

The percentage change in the body weight, food intake, and water intake was observed to be significantly reduced (*P* < 0.05) (*P* < 0.001) (*P* < 0.01) in the TDF group when compared to the normal group, as shown in Figure 7. This was significantly reversed in the AP + TDF (*P* < 0.01), AC200+TDF-, AC400+TDF-, VN200+TDF-, and VN400+TDF (*P* < 0.05)-treated groups compared to the TDF group, the details of which are provided in Supplementary Material File S7.

#### 5.3.2 Organ weight

##### 5.3.2.1 Liver and kidney

**FIGURE 8:**
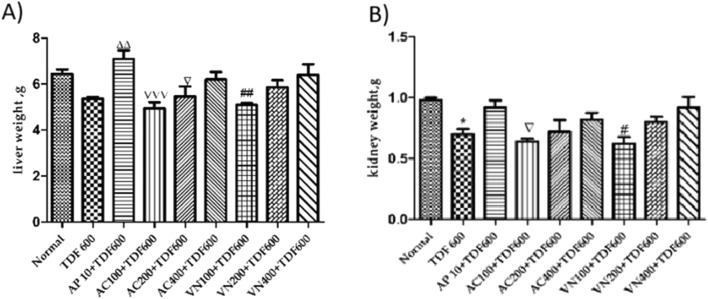
Effects of AP, AC, and VN on **(A)** liver and **(B)** kidney weight. All the values are expressed as mean ± SEM (n = 5). One-way analysis pf variance (ANOVA) followed by Tukey’s test. Statistical significance is indicated as *p < 0.05, **p < 0.01, and ***p < 0.001 compared to normal; Δp < 0.05, ΔΔp < 0.01, and ΔΔΔp < 0.001 compared to TDF.

In the TDF-treated group, a significant decrease in kidney weight was observed (*P* < 0.05), while no statistically significant change was noted in liver weight. However, a slight reduction in liver weight was observed when compared to that in the normal group. In contrast, the AP + TDF group showed a significant improvement in the liver and kidney weight (*P* < 0.01) compared to the TDF-treated group, as illustrated in Figure 8 and detailed in Supplementary Material File S8.

#### 5.3.3 Estimation of urine parameters

The TDF-treated group showed a rise in albumin level (+++) compared to the normal group; in addition, the AP-treated group had a moderate amount of albumin (++) in the urine, and some traces of albumin were also present in all the treatment groups. Furthermore, no bile pigment and bile salt were detected in all the nine groups (Supplementary Material File S9).

#### 5.3.4 Estimation of liver markers

**FIGURE 9:**
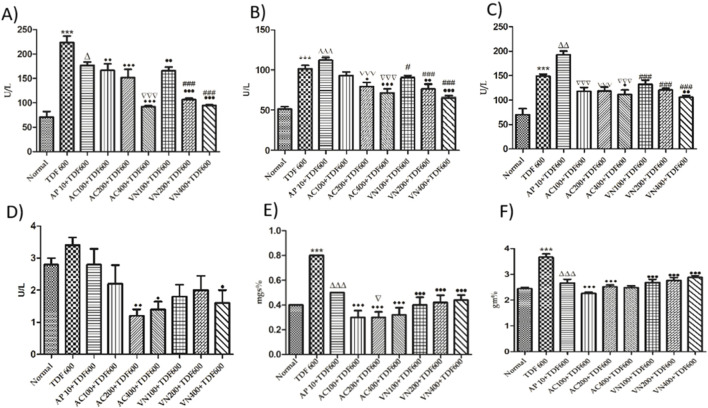
Effects of AP, AC, and VN on liver markers: **(A)** ALP, **(B)** ALT, **(C)** AST, **(D)** bilirubin, **(E)** albumin, and **(F)** GGT levels in the serum. All the values are expressed as mean ± SEM (n = 5). One-way analysis of variance (ANOVA), followed by Tukey’s test. Statistical significance is indicated as ***p < 0.001 vs. normal, ΔΔΔp < 0.001, Δp < 0.05 vs. TDF; ^•••^p < 0.001, ^••^p < 0.01, and ^•^p < 0.05 vs. TDF; ###p < 0.001, ##p < 0.01, and #p < 0.05 vs. AP.

As shown in Figure 9, a significant increase (P < 0.001) in AST, ALT, ALB, ALP, bilirubin, and GGT levels was observed in the TDF-treated group compared to the normal group. The AP + TDF group showed a significant increase in ALT (P < 0.001) and AST (P < 0.01) levels compared to the TDF group. However, a significant decrease in ALB and bilirubin (P < 0.001) and ALP (P < 0.05) was observed compared to the TDF group. Furthermore, all treatment groups exhibited a significant reduction in AST, ALT, and ALP (P < 0.001), as well as GGT (P < 0.01), compared to the TDF group. Additionally, compared to the AP + TDF group, treatment groups showed a significant decrease in AST and ALT levels. These findings are detailed in Supplementary Material File S10.

#### 5.3.5 Estimation of kidney parameters

**FIGURE 10:**
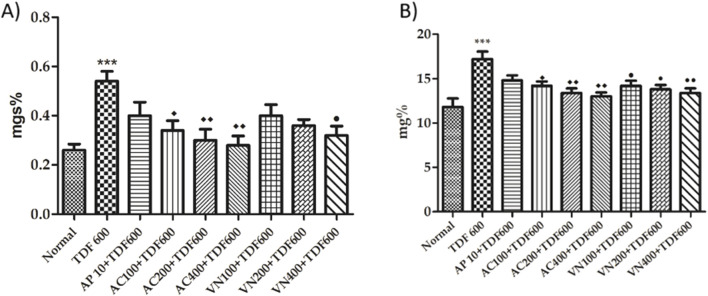
Effects of AP, AC, and VN on kidney markers: **(A)** creatinine and **(B)** BUN levels in the serum. All the values are expressed as mean ± SEM (n = 5). One-way analysis pf variance (ANOVA) followed by Tukey’s test. Statistical significance is indicated as ***p < 0.001 vs. normal, ^♦♦♦^p < 0.001, ^♦♦^p < 0.01, and ^♦^p < 0.05 vs. TDF.

A significant increase (*P* < 0.001) in creatinine and blood urea nitrogen levels in the TDF group when compared to the normal group was observed, as shown in Figure 10; in addition, there was a significant decrease (*P* < 0.01) in the creatinine level in the AC200 + TDF, AC400 + TDF, VN400 + TDF (*P* < 0.05), VN100 + TDF, VN200 + TDF, and AC100 + TDF groups when compared to the TDF group, as detailed in Supplementary Material File S11.

#### 5.3.6 Serum electrolyte

**FIGURE 11:**
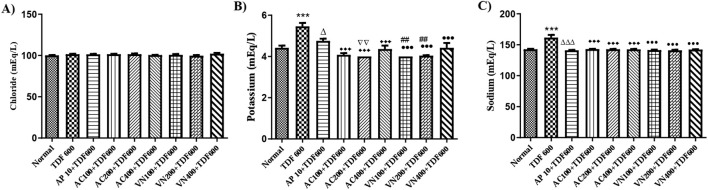
Effects of AP, AC, and VN on electrolyte levels: **(A)** chloride, **(B)** potassium, and **(C)** sodium levels in the serum. All the values are expressed as mean ± SEM (n = 5). One-way analysis of variance (ANOVA) followed by Tukey’s test. Statistical significance is indicated as ***p < 0.001 vs. normal, ΔΔΔp < 0.001 and Δp < 0.05 vs. TDF; ^⋇⋇⋇^p < 0.001, ^⋇⋇^p < 0.01, and ^⋇^p < 0.05 vs. TDF; ##p < 0.01 vs. AP + TDF.

As shown in Figure 11, there was a significant increase (*P* < 0.001) in K+ and Na + levels, but there is no significant change in the Cl- level in the TDF-treated group when compared to the normal group. A significant decrease (*P* < 0.001) in Na + and (*P* < 0.05) K+ levels in the AP + TDF group was seen when compared to the TDF group, and in all the treatment groups, there is a significant decrease (*P* < 0.001) in K+ and Na + levels when compared to the TDF group and a significant decrease (*P* < 0.01) in the K+ level when compared to the AP + TDF group, as detailed in Supplementary Material File S12.

#### 5.3.7 Enzymatic and non-enzymatic antioxidant biomarkers in liver homogenates

**FIGURE 12:**
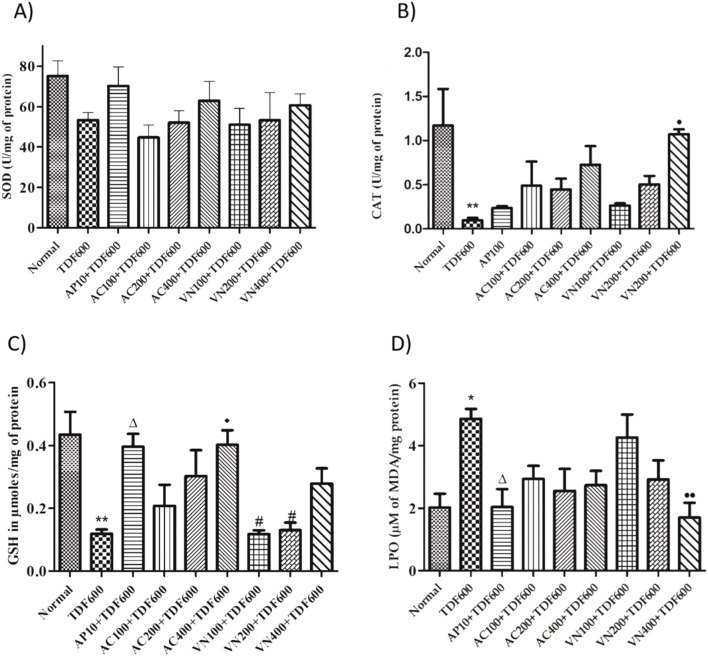
Effects of AP, AC, and VN on non-enzymatic antioxidant biomarkers in the liver homogenate: **(A)** SOD, **(B)** CAT, **(C)** GSH, and **(D)** LPO. All the values are expressed as mean ± SEM (n = 5). One-way analysis of variance (ANOVA) followed by Tukey’s test. Statistical significance is indicated as *p < 0.01 and p < 0.05 vs. Normal; Δp < 0.05 vs. TDF; ^••^p < 0.01 and ^•^p < 0.05 vs. TDF.

In the TDF-treated group, a significant decrease in catalase activity (*P* < 0.01) and GSH levels (*P* < 0.05) was observed, with no significant change in SOD levels. However, a significant increase in LPO levels (*P* < 0.01) was noted when compared to the normal group. In the AP + TDF group, a significant increase in GSH levels (*P* < 0.01) and a significant decrease in LPO levels (*P* < 0.05) were observed compared to the TDF-treated group. Additionally, in the VN400 + TDF group, GSH levels showed a significant increase (*P* < 0.05) compared to the TDF-treated group, along with a significant decrease in LPO levels (*P* < 0.01) when compared to the AP + TDF group. These findings are presented in Figure 12 and Supplementary Material File S13.

#### 5.3.8 Enzymatic and non-enzymatic antioxidant biomarkers in kidney homogenates

**FIGURE 13:**
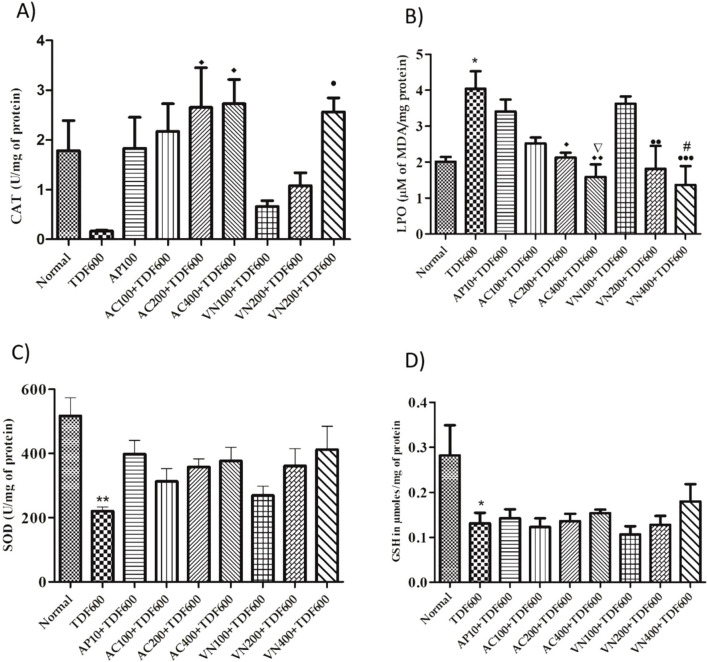
Effects of AP, AC, and VN on non-enzymatic antioxidant biomarkers in kidney homogenates: **(A)** CAT, **(B)** LPO, **(C)** SOD, and **(D)** GSH. All the values are expressed as mean ± SEM (n = 5). One-way analysis of variance (ANOVA) followed by Tukey’s test. Statistical significance is indicated as *p < 0.01 and p < 0.05 vs. Normal; Δp < 0.05 vs. TDF; ^••^p < 0.01 and ^•^p < 0.05 vs. TDF.

In the TDF-treated group, a significant decrease in SOD levels (*P* < 0.01) and GSH levels (*P* < 0.05) was observed, with no significant change in catalase levels. Additionally, a significant increase in LPO levels (*P* < 0.05) was noted when compared to the normal group. In the AC200, AC400, and VN400 groups, catalase levels showed a significant increase (*P* < 0.05), with further significant increases observed in the AC400 and VN200 groups (*P* < 0.01) and in the AC200 and VN400 groups (*P* < 0.05) when compared to the TDF-treated group. Furthermore, a significant decrease in LPO levels (*P* < 0.05) was observed in the AC400 and VN400 groups when compared to the AP + TDF group. These results are presented in Figure 13 and Supplementary Material File S14.

#### 5.3.9 Histopathological analysis

##### 5.3.9.1 Histopathology of the liver

**FIGURE 14:**
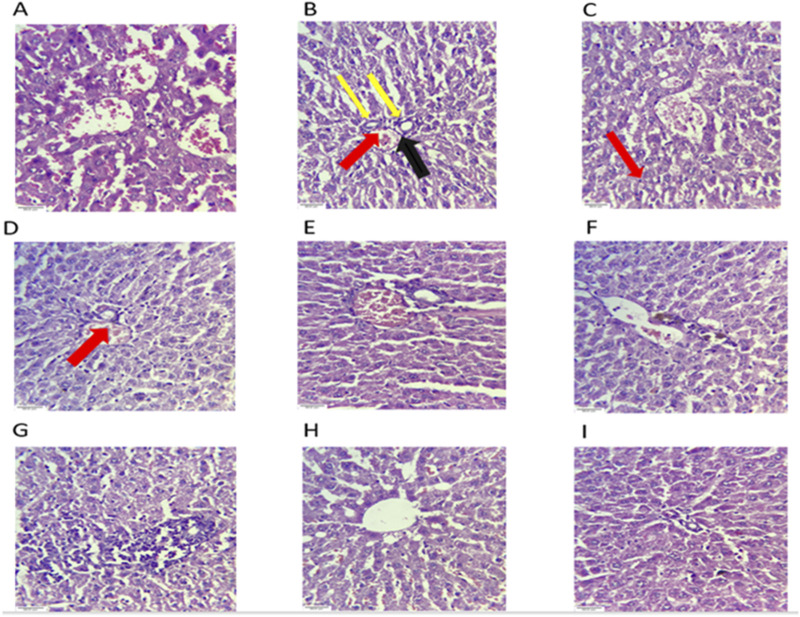
Effects of AP, AC, and VN on liver histology. Photograph of liver sections of different treatment groups stained with hematoxylin and eosin. Plates at ×40 magnification, **(A)** normal, **(B)** TDF, **(C)** AP10+TDF, **(D)** AC100+TDF, **(E)** AC200+TDF, **(F)** AC400+TDF, **(G)** VN100+TDF600, **(H)** VN200+TDF, and **(I)** VN400+TDF. The group **(B)** showed venous congestion (black arrow), ballooning degeneration (yellow arrow), and **(C)** sinusoidal congestion (red arrow).

Histopathological examination of liver sections from the TDF-treated group revealed distinct morphological alterations characterized by central venous congestion (black arrow), ballooning degeneration of hepatocytes (yellow arrow), and sinusoidal congestion (red arrow), indicative of hepatocellular injury and vascular abnormalities. In contrast, animals treated with plant extracts demonstrated a marked reduction in hepatic lesions, showing a near-normal architecture with reduced sinusoidal and venous congestion and minimal hepatocellular ballooning to varying degrees across the treatment groups (Figure 14).

##### 5.3.9.2 Histopathology of the kidney

**FIGURE 15:**
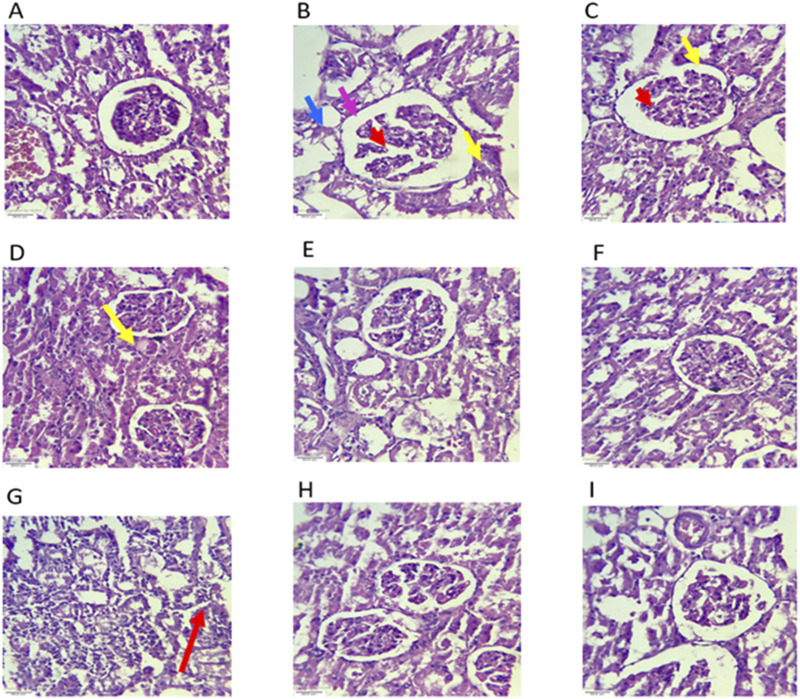
Effects of AP, AC, and VN on kidney histology. Photograph of kidney sections of different treatment groups stained with hematoxylin and eosin. Plates at ×40 magnification, **(A)** normal, **(B)** TDF, **(C)** AP10+TDF, **(D)** AC100+TDF, **(E)** AC200+TDF, **(F)** AC400+TDF, **(G)** VN100+TDF600, **(H)** VN200+TDF, and **(I)** VN400+TDF. The group **(B)** showed tubular congestion (yellow arrow), glomerular congestion (red arrow), cytoplasmic vacuoles (blue), and tubular degeneration (pink arrow).

Microscopic evaluation of the renal tissue (Figure 15) from the TDF-treated group showed severe tubular congestion (yellow arrow), glomerular congestion (red arrow), cytoplasmic vacuolization of tubular epithelial cells (blue arrow), and tubular degeneration (pink arrow), which are consistent with nephrotoxic injury. Conversely, kidney sections from the extract-treated groups showed substantial histological improvement, with a noticeable reduction in tubular and glomerular congestion, diminished cytoplasmic vacuolization, and attenuation of peritubular inflammatory infiltrates, indicating the protective effects of the plant extracts against TDF-induced renal damage.

## 6 Discussion

The present study elucidates the protective effects of *A. cepa* bulb and *V. negundo* root extracts against TDF-induced toxicities in rats. Treatment with these extracts significantly ameliorated hepatic and renal toxicities, as evidenced by the reversal of elevated biochemical markers, restoration of serum electrolyte balance, and mitigation of histopathological alterations observed in TDF-administered animals. Their therapeutic potential was further supported by significant XO-inhibitory, antioxidant, and NO scavenging activities, contributing to the overall hepatorenal protective effects. These findings highlight the multifaceted role of *A. cepa* and *V. negundo* as potential natural adjuncts for attenuating drug-induced organ toxicity.

TDF is a widely used first-line antiviral agent for managing HIV and CHB infections (Kohler et al., 2009). However, prolonged TDF administration is strongly associated with nephrotoxicity, hepatic dysfunction, osteomalacia, and other metabolic disturbances. Adverse outcomes include tubular cell injury, mitochondrial dysfunction, inflammation, and Fanconi syndrome (Libório et al., 2008). Animal and clinical studies have consistently identified TDF as a proximal tubular toxin (Lee and Marosok, 2003).

The present computational study revealed that phytocompounds from *A. cepa* and *V. negundo* showed strong binding affinity for XO, surpassing many other plant-derived compounds. These findings indicate their potential role in mitigating oxidative damage by suppressing XO-mediated ROS generation. In accordance with previous reports, with allopurinol as a potent XO inhibitor (Duong et al., 2017), the phytochemicals from these plants exhibited comparable or enhanced binding interactions with XO. As XO inhibition prevents ROS overproduction and subsequent mitochondrial injury, these compounds likely interrupt the cascade, leading to proximal tubular dysfunction, Fanconi syndrome, and oxidative stress-driven renal injury.

Consistent with the docking results, *in vitro* XO inhibition assays demonstrated that *V. negundo* root and seed hydroalcoholic extracts exhibited the highest inhibition (98.97% and 91.22%, respectively), while *A. cepa* bulb and skin methanolic extracts showed comparable inhibition (97.45% and 95.12% at 500 μg/mL). Furthermore, both extracts exhibited robust DPPH and NO radical scavenging activities, confirming their antioxidant potential and relevance in mitigating mitochondrial oxidative stress.

Co-treatment with these extracts also led to a significant improvement in the physical and physiological parameters, including body weight, food, and water intake, compared to the TDF group. Notably, increased relative liver and kidney weights in the extract-treated groups further support their prophylactic potential in preventing TDF-induced organ damage.

Biochemical analysis revealed that TDF administration markedly elevated ALP, ALT, AST, bilirubin, albumin, GGT, creatinine, and BUN levels, which are key indicators of hepatic and renal dysfunction. Similar results were also reported previously by Lai et al. (2025). These elevations reflect TDF-induced oxidative damage, wherein superoxide anions impair mitochondrial function and exacerbate liver injury (Duan et al., 2021; Vazi et al., 2023). The extract-treated groups showed significant reductions in these parameters, confirming their hepatorenal protective efficacy. Additionally, the reversal of increased LPO and restoration of GSH, SOD, and CAT levels highlight the antioxidant-mediated protection conferred by *A. cepa* and *V. negundo* extracts.

The histopathological findings further corroborate the biochemical and molecular observations, indicating that TDF-induced hepatic and renal damage is primarily mediated through oxidative stress, mitochondrial dysfunction, and XO-driven free radical generation. The ballooning degeneration of hepatocytes, vascular congestion, and tubular necrosis observed in the TDF-treated group are typical manifestations of drug-induced oxidative injury (Tatar et al., 2020). However, animals receiving co-treatment with XO-inhibiting plant extracts showed a notable restoration of normal tissue architecture, characterized by reduced inflammatory infiltration, attenuation of vascular congestion, and preservation of cellular morphology. These improvements indicate that the antioxidant, anti-inflammatory, and XO-inhibitory properties of the phytoconstituents effectively mitigated TDF-induced oxidative and inflammatory responses, thereby conferring hepatorenal protection. Mechanistically, TDF-induced ROS generation disrupts hepatocellular and renal homeostasis, leading to oxidative and nitrosative stress, mitochondrial damage, and altered signaling pathways (Lee et al., 2021). These alterations elevate renal interstitial pressure and vascular resistance, thus impairing glomerular filtration and promoting renal dysfunction. The observed biochemical and histopathological improvements in the extract-treated groups indicate that *A. cepa* and *V. negundo* effectively attenuate oxidative stress-mediated organ injury through antioxidant and XO-inhibitory mechanisms.

Overall, these results indicate that *A. cepa* and *V. negundo* extracts show significant hepatorenal protective effects against TDF-induced toxicity through a combination of antioxidant, anti-inflammatory, and XO-inhibitory actions, underscoring their potential application in adjunctive therapy for antiviral drug-induced organ damage.

## 7 Conclusion

The present study demonstrated that XO-inhibiting phytoconstituents from *A. cepa* and *V. negundo* show strong XO-inhibitory activity in both computational and *in vitro* evaluations, thereby conferring significant protection against TDF-induced hepatorenal toxicities. Furthermore, *in vivo* findings revealed that concomitant administration of selected extracts from these plants significantly mitigated TDF-induced renal and hepatic toxicity, as evidenced by improvements in the physiological parameters (body weight and food and water intake) and biochemical markers (AST, ALT, creatinine, BUN, SOD, GSH, catalase, and LPO). Collectively, these results indicate that *A. cepa* bulb and *V. negundo* root extracts possess promising hepatorenal protective potential through XO inhibition and antioxidant mechanisms. However, further pharmacokinetic and clinical studies are warranted to validate their safety and efficacy in human trials to explore their therapeutic potential in managing drug-induced organ toxicities in chronic diseases such as HIV and CHB.

## Data Availability

The datasets presented in this study can be found in online repositories. The names of the repository/repositories and accession number(s) can be found in the article/Supplementary Material.
